# Peritoneal dissemination of appendiceal goblet cell adenocarcinoma mimicking white pus caused by peritonitis following appendicitis: an instructive case report

**DOI:** 10.1186/s40792-024-01847-4

**Published:** 2024-02-22

**Authors:** Keigo Nakashima, Masakazu Hashimoto, Yoshihito Kitamura, Makoto Shinohara, Mizuki Yamaguchi, Michinori Hamaoka, Masashi Miguchi, Toshihiro Misumi, Nobuaki Fujikuni, Satoshi Ikeda, Yasuhiro Matsugu, Takashi Nishisaka, Hideki Nakahara

**Affiliations:** 1 Department of Gastroenterological Surgery, Hiroshima Prefectural Hospital, 1-5-54 Ujinakannda, Minami-ku, Hiroshima, 734-8530 Japan; 2 Department of Pathology and Laboratory Medicine, Hiroshima Prefectural Hospital, Hiroshima, Japan

**Keywords:** Appendiceal tumor, Goblet cell adenocarcinoma, Peritoneal dissemination

## Abstract

**Background:**

Goblet cell adenocarcinoma is an extremely rare tumor in which the same cells exhibit both mucinous and neuroendocrine differentiation. It is considered more aggressive compared to conventional carcinoids and more likely to cause metastasis.

**Case presentation:**

We report a case of goblet cell adenocarcinoma with peritoneal metastases. A 62-year-old man underwent appendectomy for acute appendicitis. Intraoperatively, inflammatory white pus and a small amount of dirty ascites were observed in the lower abdomen with severely inflamed appendix. Histopathological examination of the specimen collected during appendectomy revealed goblet cell adenocarcinoma with a positive surgical margin. One month later, additional ileal resection was planned. Laparoscopic examination revealed disseminated nodules throughout the abdominal cavity. Therefore, the patient underwent resection of the peritoneal nodules. The peritoneal specimens confirmed the histopathological findings. Thus we diagnosed the patient with peritoneal dissemination of appendiceal goblet cell adenocarcinoma.

**Conclusions:**

In cases wherein white pus is observed during surgery for acute appendicitis, considering the possibility of dissemination, collecting samples for histopathological examination, and initiating early treatment are crucial.

## Background

Goblet cell adenocarcinoma (GCA), formerly known as goblet cell carcinoma, is a rare appendiceal tumor with amphicrine differentiation; it has an incidence of 0.01–0.05 per 100,000 persons annually and accounts for approximately 15% of all appendiceal neoplasms [1–3]. Appendiceal tumors tend to exhibit peritoneal dissemination; it has been suggested that, among the appendiceal tumors, GCA is more likely to develop peritoneal dissemination [4, 5]. We present a case of GCA with peritoneal dissemination that was preoperatively diagnosed as appendicitis.

## Case presentation

**Fig. 1:**
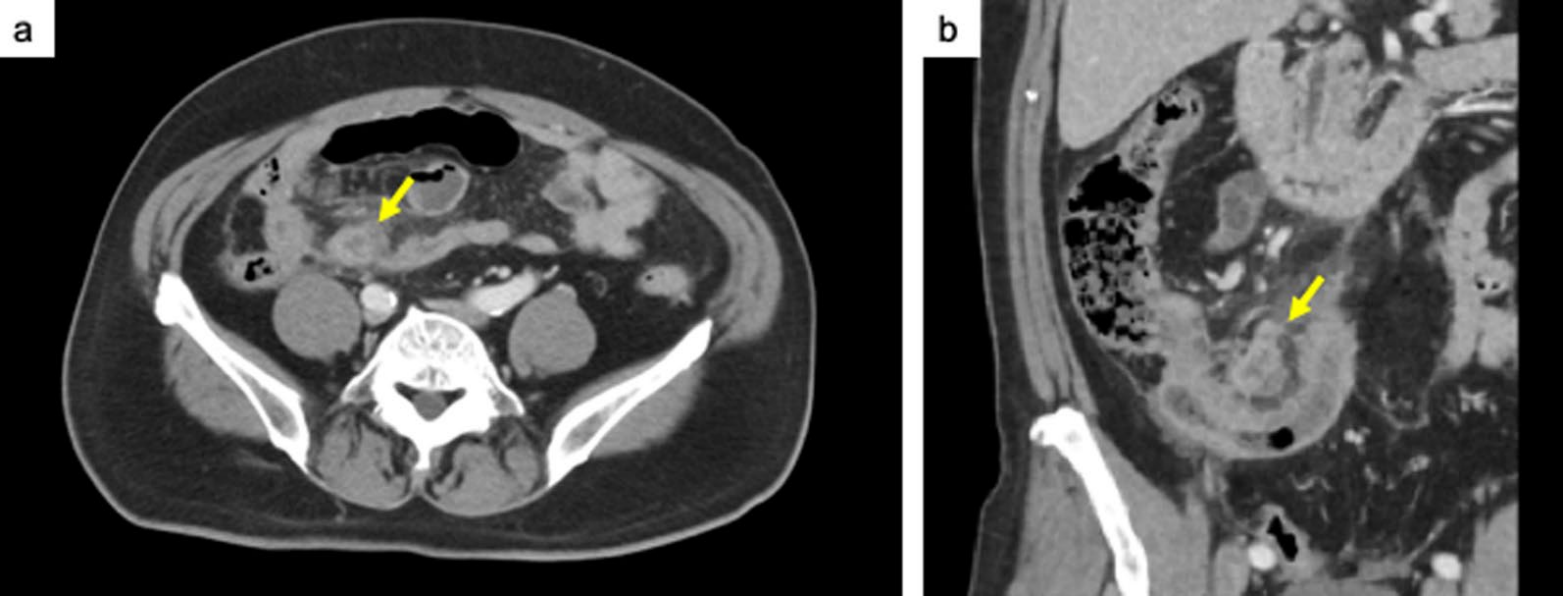
Abdominal computed tomography reveals acute appendicitis with swelling at the tip of the appendix and surrounding edema (yellow arrow)

**Fig. 2:**
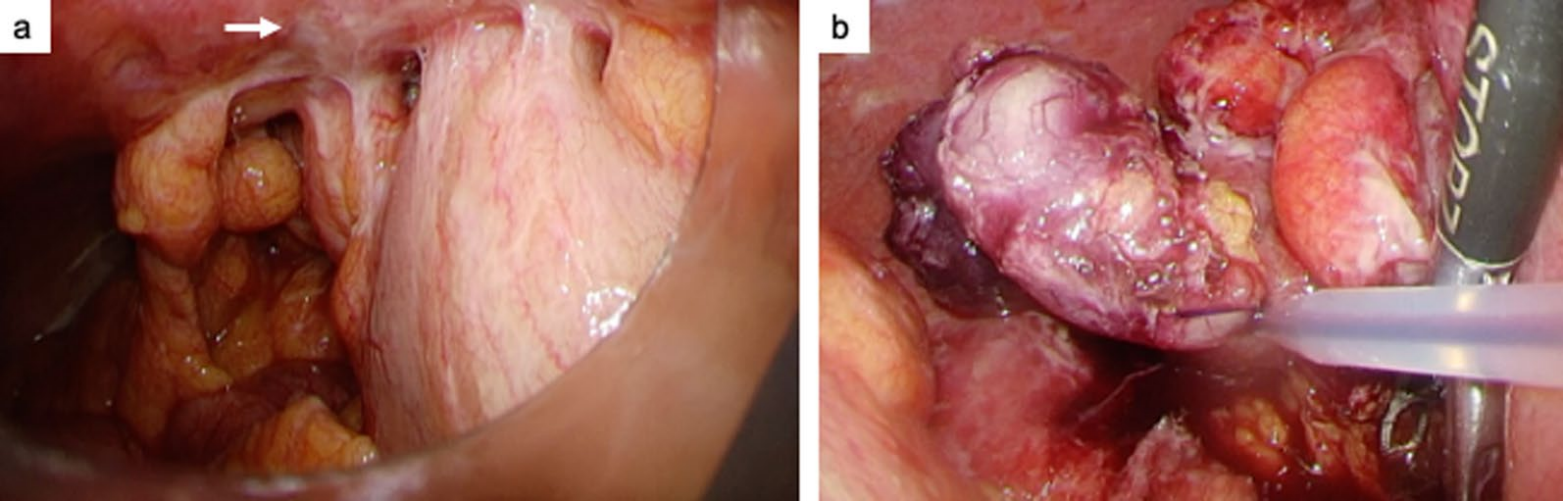
**a** Intraoperatively, inflammatory white pus (white arrow) and adhesions are apparent in the lower abdominal region. **b** Appendectomy is completed using two endoloops at the root of the appendix

A 62-year-old man with lower abdominal pain was admitted to our hospital. Fever and gastrointestinal symptoms such as vomiting and diarrhea were not observed. The patient had no history of surgery or significant family medical history. Physical examination indicated a soft abdomen with tenderness in the right lower quadrant. Laboratory test results revealed mild leukocytosis (11,400/μL) and increased C-reactive protein levels (22.65 mg/dL). Abdominal computed tomography revealed swelling at the tip of the appendix with surrounding inflammation, leading to the diagnosis of acute appendicitis (Fig. 1). Laparoscopic appendectomy was performed on the same day. Intraoperatively, inflammatory white pus was observed in the lower abdominal region involving the small bowel mesenterium, abdominal peritoneum, and omentum (Fig. 2a). The appendix was severely inflamed and adhered to the surrounding small bowel. Additionally, a small amount of dirty ascites was observed. The adhesions were gently mobilized. Thereafter, appendectomy was performed using two endoloops® PDS® at the root of the appendix (Fig. 2b). The patient experienced good postoperative recovery and was discharged in good condition on postoperative day seven.

**Fig. 3:**
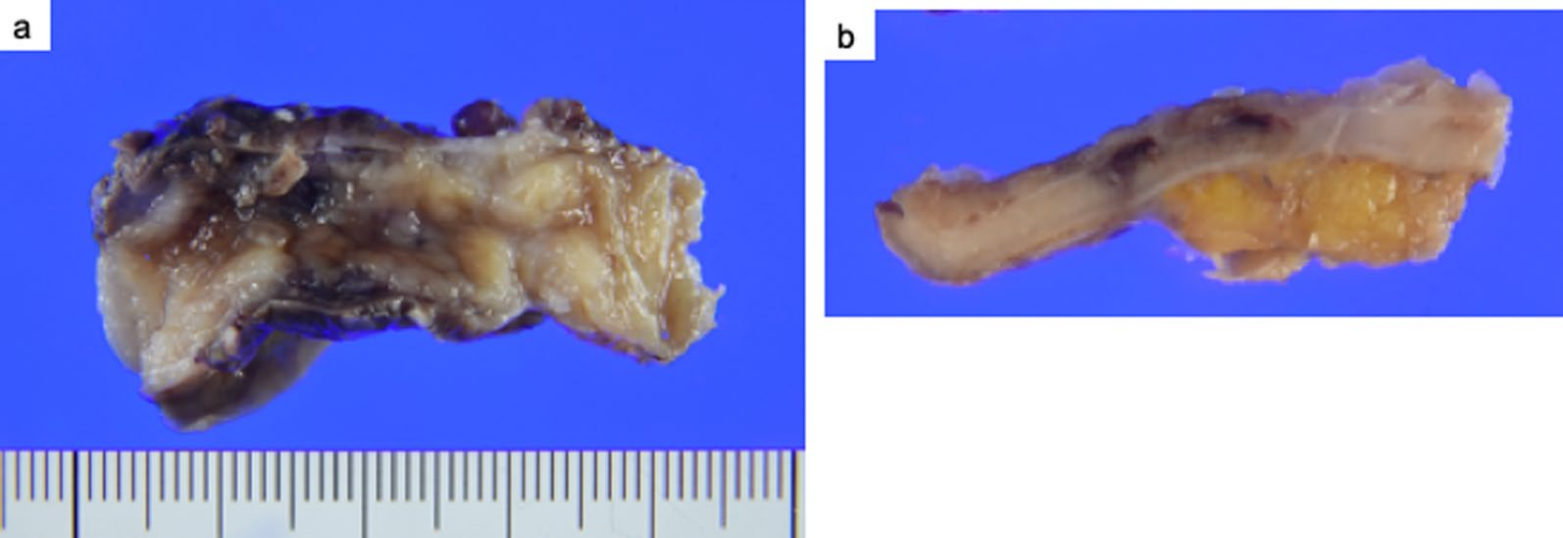
**a**, **b** Macroscopic view of the resected specimen, which is a 40- × 11- × 6-mm lesion with a positive margin

**Fig. 4:**
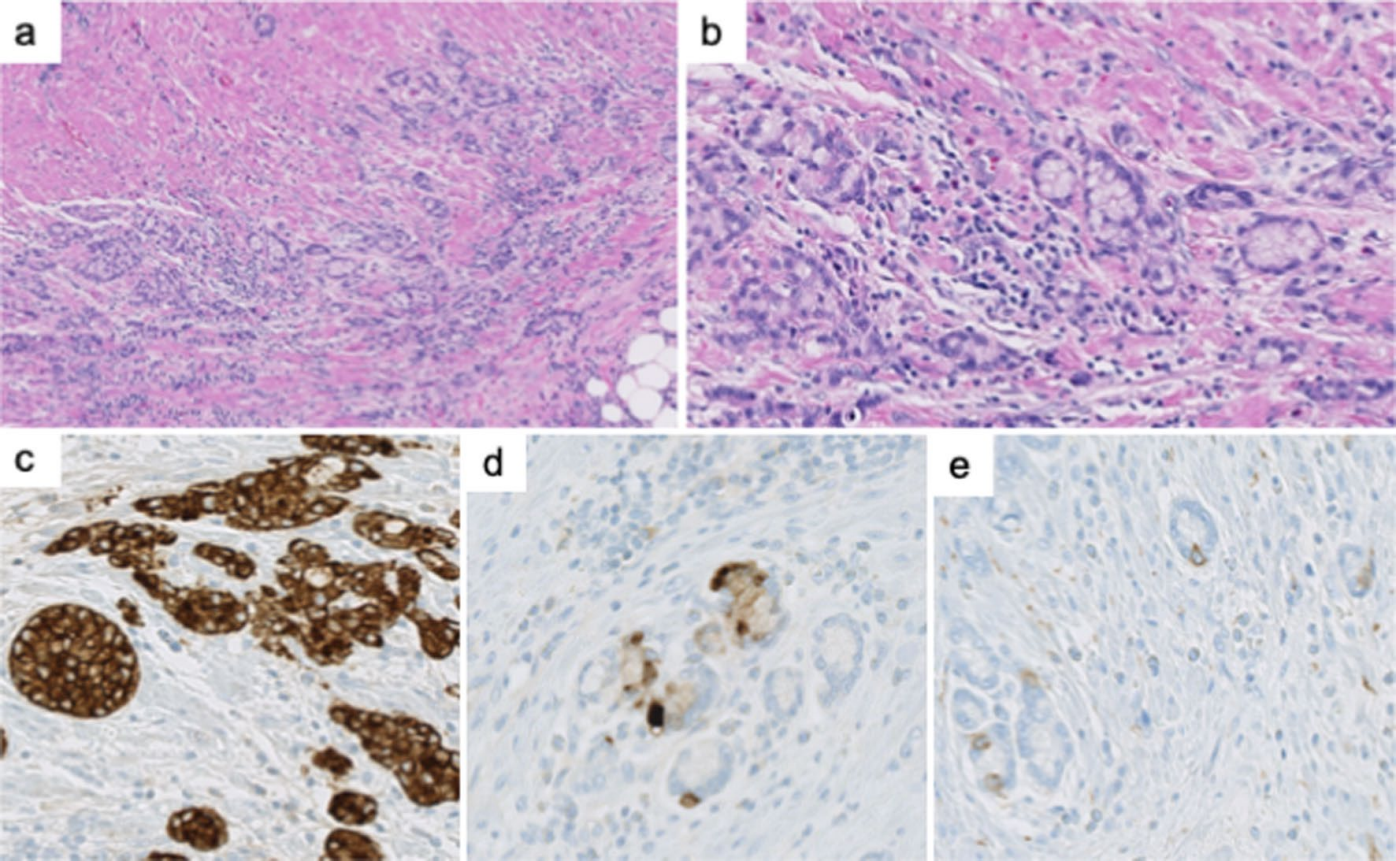
Histopathological findings of the appendix lesion. **a**, **b** Hematoxylin and eosin (H&E) staining shows tumor cells with small lumina comprising goblet-like cells, and some tumor cell clusters lack lumina and appear as small groups of cohesive goblet-like cells. Low-grade patterns comprised less than 50%. **c**–**e** Immunohistochemical staining indicates that the tumor cells are positive for cytokeratin AE_1_/AE_3_ (**c**), and various number of endocrine cells are positive for chromogranin A (**d**), and synaptophysin (**e**)

**Fig. 5:**
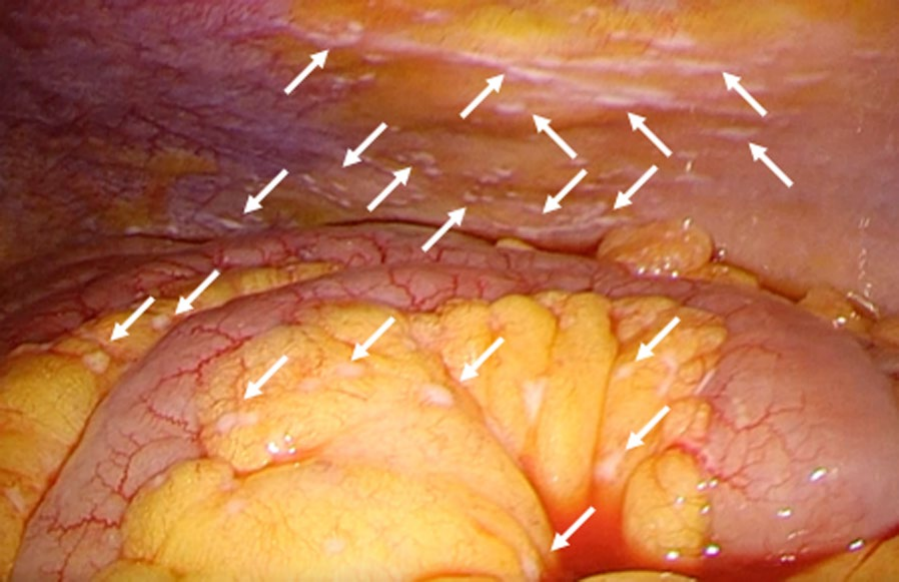
Intraoperative laparoscopic findings. White nodules are seen on the mesentery (white arrow)

The histopathological examination of the specimen collected during appendectomy revealed GCA (according to the 5th edition of the World Health Organization classification). The macroscopic examination revealed that the resected appendix had a 40- × 11- × 6-mm lesion with a positive proximal resection margin (Fig. 3). The histopathological examination indicated that the tumor contained small lumina comprising goblet-like cells, and some tumor cell clusters lacked lumina and displayed small groups of cohesive goblet-like cells (Fig. 4a, b). Low-grade patterns comprised less than 50%, so we categorized this case as grade G3. On the immunohistochemical examination, the tumor cells were positive for cytokeratin AE_1_/AE_3_, and various number of endocrine cells were positive for synaptophysin, and chromogranin A (Fig. 4c–e). Since the histopathological examination revealed GCA and pT4a, an additional ileocecal resection was planned. Additional laboratory test results showed carcinoembryonic antigen and carbohydrate antigen 19–9 levels of 1.1 ng/mL and 13 U/mL, respectively. Laparoscopy was performed 1 month after appendectomy, and white nodules were observed throughout the abdominal cavity: raising the suspicion of peritoneal dissemination (Fig. 5). Using the intraoperative rapid pathological diagnosis method, these nodules were diagnosed as adenocarcinomas and considered inoperable. The Peritoneal Carcinomatosis Index was considered to be 18. Postoperative positron emission tomography showed scattered thickening of the pelvic peritoneum but no accumulation. The patient received chemotherapy and remained alive and progression-free for 6 months after surgery.

## Discussion

Two important clinical issues were identified during this experience. First, the patient was initially considered to have typical symptoms of appendicitis requiring emergency surgery. However, a postoperative pathological examination revealed the presence of GCA. Second, white plaques were observed during emergency surgery and later identified as peritoneal dissemination during a second surgery for GCA.

During emergency surgery for appendicitis, it was found that what was mistaken for white pus could have been peritoneal dissemination. Although there are no reported cases of peritoneal dissemination misdiagnosed as white pus during emergency surgery for acute appendicitis, it is possible for such incidents to occur in clinical practice. Peritoneal dissemination is relatively common in tumors such as appendiceal cancer and GCA, with pseudomyxoma peritonei occurring frequently in mucinous carcinoma [6]. Moreover, due to the combination of appendiceal inflammation and histological characteristics such as a thin muscular layer and the propensity for tumor invasion to reach the serosa, appendiceal cancer has a higher incidence of perforation. Therefore, peritoneal dissemination is typically observed in approximately 5% of all colorectal cancer cases and characterized by a high incidence (20%) of appendiceal cancer [7, 8]. Based on these findings, peritoneal metastasis was considered in our case and surgery was performed accordingly.

Peritoneal dissemination could not be identified for three reasons. First, we diagnosed diffuse or localized peritonitis due to appendiceal perforation before surgery. This factor could have led to a positive resection margin, and we considered the possibility of malignancy from the root of the appendiceal tissue during resection. However, it was finally recognized as an extension of appendicitis. Second, the white pus primarily adhered to the peritoneum of the abdominal wall, with less noticeable attachment to the mesenteric or omental surface. Third, compared to open surgery, lack of tactile sensation during laparoscopic surgery may have led to difficulty in identifying peritoneal dissemination. The lesson learned from our case is that appendiceal cancer could be prone to peritoneal metastasis and require careful observation. A rapid histopathological diagnosis was challenging because emergency surgery was performed late at night. However, when peritoneal dissemination is suspected, it is advisable to collect a sample of white pus for histopathological examination.

Appendiceal neoplasms are rarely diagnosed preoperatively or during emergency surgery. The frequency of appendiceal cancer in cases of appendicitis is 0.78–1.4% [9–12]. In the present case, preoperative computed tomography revealed appendiceal enlargement and surrounding fluid accumulation, leading to the diagnosis of acute appendicitis with perforation. Cystic lesions and tumor formation have been reported as the imaging characteristics of appendiceal cancer. An appendix larger than 15 mm with thickened or irregular walls on multidetector computed tomography or magnetic resonance imaging should incur suspicion of neoplasia [13]. However, many studies have highlighted the difficulty in determining the preoperative diagnosis. At our facility, during the past 5 years, we have performed 351 appendectomies for appendicitis. Two (0.6%) of those patients were diagnosed with appendiceal cancer. None of the patients showed any evidence of appendiceal cancer preoperatively. In cases wherein appendiceal cancer is diagnosed histopathologically, quickly reviewing the surgical field in video recordings of appendectomy and not overlook signs of dissemination is important.

The treatment of GCA is reported in the multidisciplinary guidelines of the Chicago Consensus Working Group [14]. Although chemotherapy has not yet been established, because the existing evidence shows limited efficacy, standard chemotherapy, which is used for other colorectal cancers, for a total of 6 months is usually recommended [14, 15]. A multicenter retrospective study of Japanese appendiceal GCA, including 20 cases, showed that the treatment is mainly similar to chemotherapy for colon cancer [16]. Intraperitoneal chemotherapy has been used for treating peritoneal metastases of appendiceal origin [17]. In our case, systemic chemotherapy comprising capecitabine, oxaliplatin, and bevacizumab was administered, and the disease remained stable during the 6-month postoperative period. More studies are necessary to further assess improvements in survival rates associated with GCA.

## Conclusions

In our case, peritoneal dissemination of GCA mimicked white pus due to diffuse peritonitis following acute appendicitis. In cases wherein white pus is observed during emergency surgery for acute appendicitis, considering the possibility of dissemination, collecting samples for histopathological examination, and initiating early treatment might be crucial.

## Data Availability

Not applicable.

## Abbreviation

GCA: Goblet cell adenocarcinoma
